# Leprosy survey among rural communities and wild armadillos from Amazonas state, Northern Brazil

**DOI:** 10.1371/journal.pone.0209491

**Published:** 2019-01-10

**Authors:** Mariane Martins Araújo Stefani, Patricia Sammarco Rosa, Mauricio Barcelos Costa, Antônio Pedro Mendes Schetinni, Igor Manhães, Maria Araci Andrade Pontes, Patricia Costa, Luciana Raquel Vincenzi Fachin, Ida Maria Foschiani Dias Batista, Marcos Virmond, Emília Pereira, Maria Lucia Fernandes Penna, Gerson Oliveira Penna

**Affiliations:** 1 Instituto de Patologia Tropical e Saúde Pública, Universidade Federal de Goiás, Goiânia, Goiás, Brazil; 2 Instituto Lauro de Souza Lima Bauru, São Paulo, Brazil; 3 Faculdade de Medicina, Universidade Federal de Goias, Goiânia, Goiás Brazil; 4 Fundação de Dermatologia Tropical e Venerologia, Alfredo da Matta, Manaus Amazonas, Brazil; 5 Departamento de Dermatologia, Universidade Federal do Rio de Janeiro, Rio de Janeiro, Rio de Janeiro, Brazil; 6 Centro de Dermatologia Dona Libânia, Fortaleza, Ceará, Brazil; 7 Secretaria Municipal de Saúde, Coari, Amazonas, Brazil; 8 Instituto de Saúde Coletiva, Universidade Federal Fluminense, Niterói, Rio de Janeiro, Brazil; 9 Centro de Medicina Tropical, Universidade de Brasília, Brasília, Distrito Federal, Brazil; CEA, FRANCE

## Abstract

There is evidence that in southern US, leprosy is a zoonosis infecting wild *Dasypus novemcinctus* armadillos but the extent of this finding is unknown. This ecological study investigated leprosy in rural communities and in wild armadillos from the Brazilian Amazon. The study area was the Mamiá Lake of Coari municipality, Amazonas State, Northern region, a hyper endemic leprosy area where residents live on subsistence farming, fishing and armadillo hunting and its meat intake are frequent. The leprosy survey was conducted in sixteen communities by a visiting team of specialists. Local partakers provided wild armadillos to investigate *M*. *leprae* infection. Volunteers had complete dermato-neurological examination by a dermatologist with expertise in leprosy diagnosis, suspect skin lesions were biopsied for histopathology (Hematoxylin-eosin/HE, Fite-Faraco/FF staining); slit skin smears were collected. Armadillos’ tissue fragments (skins, spleens, livers, lymph nodes, adrenal glands, others) were prepared for histopathology (HE/FF) and for *M*. *leprae* repetitive element-RLEP-qPCR. Among 176 volunteers, six new indeterminate leprosy cases were identified (incidence = 3.4%). Suspect skin sections and slit skin smears were negative for bacilli. Twelve wild *D*. *novemcinctus* were investigated (48 specimens/96 slides) and histopathological features of *M*. *leprae* infection were not found, except for one skin presenting unspecific inflammatory infiltrate suggestive of indeterminate leprosy. Possible traumatic neuroma, granuloma with epithelioid and Langhans cells, foreign-body granuloma were also identified. Granulomatous/non-granulomatous dermatitides were periodic-acid-Schiff/PAS negative for fungus. *M*. *leprae*-RLEP-qPCR was negative in all armadillos’ tissues; no bacillus was found in histopathology. Our survey in rural communities confirmed the high endemicity for leprosy while one armadillo was compatible with paucibacillary *M*. *leprae* infection. At least in the highly endemic rural area of Coari, in the Brazilian Amazon region where infectious sources from untreated multibacillary leprosy are abundant, *M*. *leprae* infected armadillos may not represent a major source of infection nor a significant public health concern.

## Introduction

Leprosy or Hansen's disease is a chronic contagious infection caused by *Mycobacterium leprae* that affects mostly the skin macrophages and the Schwann cells of peripheral nerves [1]. The infection of Schwann cells with *M*. *leprae* can trigger immune-inflammatory mediated mechanisms, which reduce myelin production and lead to nerve fiber damage resulting in the loss of sensitivity, disfigurement and disabilities, which are considered the hallmarks of leprosy [1, 2]. More recently it has been demonstrated that leprosy, especially diffuse lepromatous leprosy, can be caused by *M*. *lepromatosis* [2]. Despite wide implementation of multidrug therapy (MDT) more than three decades ago, many countries, such as India and Brazil still report high incidence and over 200,000 new leprosy cases have been reported globally each year [3]. Untreated multibacillary leprosy patients represent both the main shedders of bacilli by aerial route and the major cause of inter-human transmission through recurrent and close contact with susceptible hosts [1]. The majority of infected individuals do not develop disease symptoms, but depending on genetic, nutritional and immunological factors, about 10% of the exposed individuals may manifest clinical disease [4].

Although for a long period, humans have been considered the only natural hosts and the main source of *M*. *leprae*, studies from the southern United States of America (US) have indicated that zoological reservoirs also play a role in leprosy transmission [5]. Evidence is now accumulating that leprosy is a zoonosis in North America and that wild *Dasypus novemcinctus* nine banded armadillos from Texas, Louisiana and Mississippi States represent *M*. *leprae* reservoirs [5, 6]. More recently a study using genomics, histopathology and serology showed that in the British Isles, red squirrels are reservoirs for leprosy and infection was detected in clearly diseased and apparently healthy animals [7].

*M*. *leprae* is an obligate intracellular pathogen that has never been cultivated *in vitro* in axenic media. *M*. *leprae* multiplies slowly and propagates mainly in cooler body areas, such as hands and feet; experimentally infected animals such as nude mice (body temperature 32°C) and armadillos (body temperature 33^0^−35°C) are alternative sources of bacilli [8]. Armadillos have been shown to represent a better animal model for leprosy as a result of their low metabolic rate and low body temperature, compared to the mouse footpad that supports limited replication of bacilli [9, 10]. Upon infection with *M*. *leprae*, armadillos produce high bacillary load and develop clinical symptoms and pathologies, similar to the human disease, including extensive peripheral nerve involvement [11, 12]. Armadillos do not reproduce well in captivity and shortly after the discovery of their unique susceptibility to experimental infection, studies have reported natural infection with *M*. *leprae* in animals caught in the wild [13, 14]. Humans infected with *M*. *leprae* and armadillos have shared the same environment for centuries and wild armadillos have probably acquired the disease from untreated patients after the colonization of the New World, long before being used as a leprosy research model [15].

A recent study in wild armadillos from Pará state in the Brazilian Amazon showed a high rate of *M*. *leprae* infection [16]. The geographic extent of naturally *M*. *leprae* infected armadillos in the wild and the existing risks of human-to-armadillo, armadillo-to-armadillo or armadillo-to-human transmission are unknown especially in highly endemic leprosy areas where *D*. *novemcinctus* is found. In this study we investigated the existence of natural infection of armadillos with *M*. *leprae* and the possible link with human disease in Amazonas state, a hyper endemic leprosy area in the Brazilian Amazon where armadillo hunting and its meat intake are recurrent.

## Material and methods

### Study design, area and population

**Fig 1:**
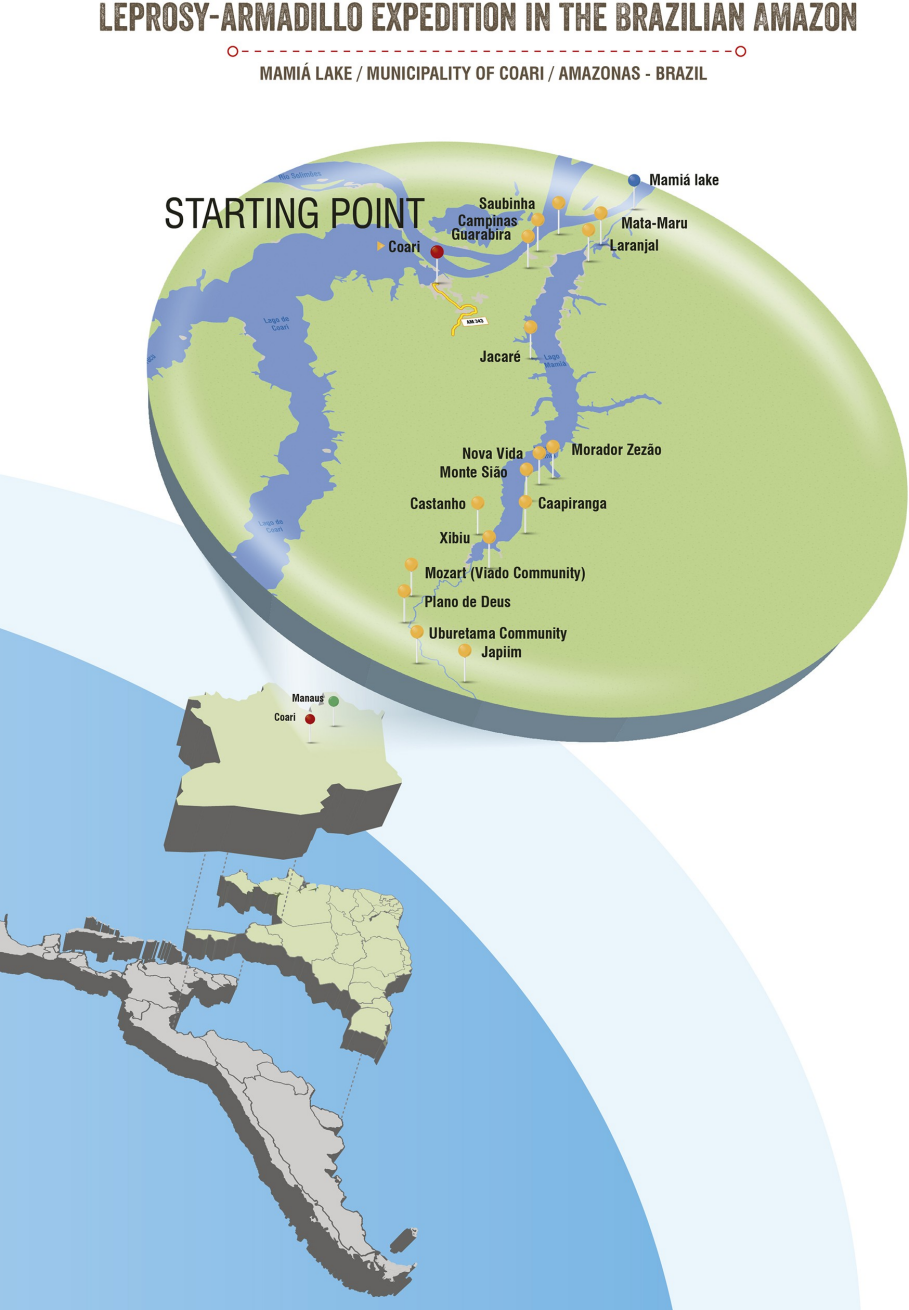
Latin America map, with emphasis to our study area: Northern Brazil, Amazonas state and the rural area of Coari municipality. The map indicates Manaus the capital of Amazonas state (green dot) and our study area Coari municipality (red dot). The highlighted area is the Mamiá Lake region (blue dot), where the fieldwork took place. The following rural communities (yellow dots) were screened for leprosy: Laranjal, Campinas, Guarabira, Saubinha, Mata Maru, Uruburetama, Plano de Deus, Mozart, Xibiu, Monte Sião, Nova Vida, Castanho, Caapiranga, Zezão, Jacaré and Japiim. Our research team conducted a specific health initiative that included an active search for new leprosy cases. Local partakers that are used to catch armadillo for its meat consumption donated biological samples for this study to investigate *M*. *leprae* infection.

This is an ecological study conducted in rural communities of a hyper endemic leprosy area of Coari municipality (coordinates: 4° 5'6''S, 63° 8'30''W), Amazonas State, Northern Brazil, located by the Solimões River, between the Mamiá and the Coari Lakes (Fig 1). Coari is situated 444 km away from Manaus (Amazonas capital) and can be reached by plane or boat (9 hours trip); over 34% of its population (80,000 inhabitants) live in rural areas. Besides an endemic area, Coari has one of the most important oil and gas provinces of Brazil (Urucu) where a 397km pipeline is under construction to carry natural gas and liquefied petroleum gas to the industrial zone of Manaus. Our study used information provided by a national program (“Strategy of Socio-environmental intelligence of Petroleum Industry of the Amazon, PIATAM”) that contains detailed description of Coari’s population and the mapping of its riverine communities. Our study area was the Mamiá Lake region of Coari, a leprosy endemic area in a far remote and isolated region with very low population density (around 1 inhabitant per km^2^) distributed in small rural communities (from less than 10, to 50 houses) (Fig 1). Local inhabitants traditionally live on subsistence farming, fishing, hunting and collecting food from the native environment. In this area, armadillo hunting and its meat intake are frequent and armadillos are usually marketed together with wild products collected from the forest. This ecologic scenario seemed ideal to investigate if wild armadillos were infected with *M*. *leprae* and if they had a role in the local leprosy epidemic.

In June 2015, a pilot expedition team went to Coari and established partnerships between the visiting research team and the local community health agents (nurses and technicians). These agents are trained in basic health education, diagnosis, treatment and follow-up of common local public health problems including leprosy (“Programa de Saúde da Família/SUS”). At this occasion, community leaders were instructed about the study and invited to provide armadillos’ samples for the study. Local inhabitants from the Mamiá lake region were oriented to take live caught wild armadillos in cages to the expedition boat where they were anesthetized (tiletamine and zolazepam, 5.0 mg/kg/I.M) before being euthanized by exsanguination.

Two months later (August 2015), a multidisciplinary research team composed of 22 members (visiting and local partakers) was assembled in Coari. Visiting researchers included a dermatologist with expertise in the clinical diagnosis of leprosy (IM), two nurses, three nurse technicians, a specialist in georeferencing and one biologist from Manaus, Amazonas. Additionally, three researchers from Lauro Souza Lima Institute (Bauru, São Paulo, Brazil) with expertise in armadillo research comprising sample collection, storage (PSR) and molecular biology (IMFDB) were also part of the team. In Coari, a local boat took the research team to the study area, the Mamiá Lake region (Fig 1).

### Leprosy survey in rural communities

**Fig 2:**
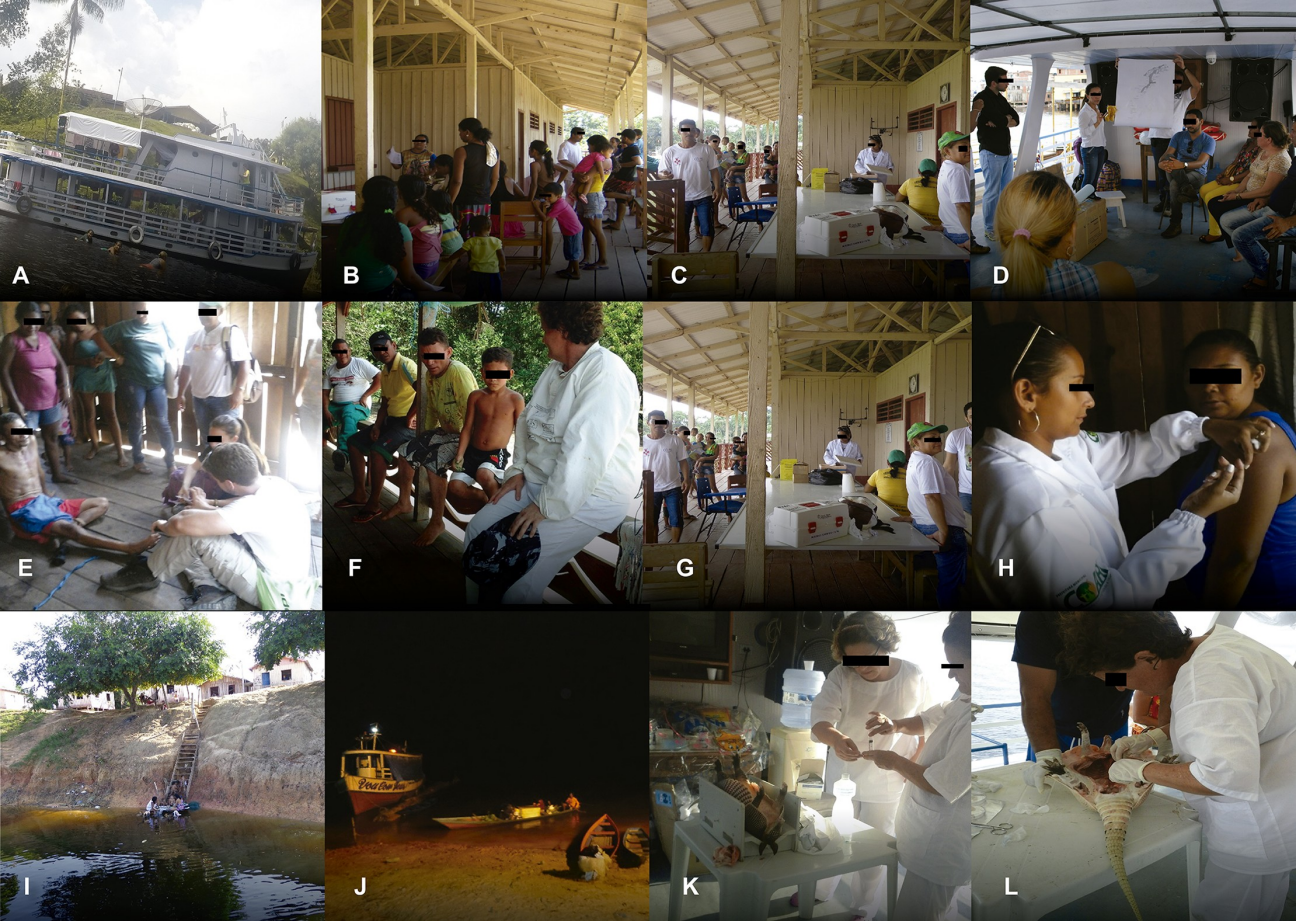
Details of leprosy survey in rural communities and in wild armadillos performed in the Mamiá Lake region, Coari municipality Amazonas, Brazil. (A): Overview of the boat used for expedition; (B), (C), (D), (E), (F), (G): Active search of new leprosy cases among inhabitants of rural communities; (H): The vaccination initiative; (I), (J): Day and night view of a village in the Mamiá Lake region included in the leprosy survey; (K), (L): Bench work at the expedition boat to collect and store biological samples of armadillos for the investigation of *M*. *leprae* infection.

The survey of new leprosy cases was conducted in the following rural communities located in the Mamiá Lake region: Laranjal, Campinas, Guarabira, Saubinha, Mata Maru, Uruburetama, Plano de Deus, Mozart, Xibiu, Monte Sião, Nova Vida, Castanho, Caapiranga, Morador Zezão, Jacaré and Japiim (Fig 1). During the expedition, the boat used to transport the research team (Fig 2A) also served for lodging and for the clinical examination of the volunteers (Fig 2B–2G). Our study included a vaccination initiative for BCG, yellow fever, hepatitis B, hepatitis A, pentavalent vaccine DTPa-VIP/Hib, triple viral/tetra viral vaccine MMR/MMRV, polio VIP/VOP, decavalent pneumococcal and meningococcal C vaccine (Fig 2H). Day and night view of a village included in the leprosy survey of riverine communities and armadillos (Fig 2I and 2J). In the expedition boat, a special area with a bench and a freezer was prepared to collect and store biological samples from armadillos (Fig 2K and 2L).

### Histopathology of human skin lesions suspect of leprosy and armadillos’ tissue samples

Following complete dermato-neurological examination by a dermatologist, skin lesions suspect of leprosy were biopsied. Skin sections were further prepared for histopathological examination after staining with hematoxylin-eosin (HE) and with Fite-Faraco (FF) for bacilli identification, using as positive controls tissue samples with high bacillary load (5+/6+). Slit skin smears were collected for bacilli identification.

For the armadillo study, tissue fragments of skins, spleens, livers and lymph nodes, adrenal glands and other organs (ovary and fallopian tubes) were preserved in 70% alcohol, 10% buffered formalin and further prepared for histopathological examination after staining with HE and FF. Two pathology experts performed the histopathological examination blindly and independently. Animal tissues presenting granulomas or non-granulomatous dermatitis were also examined for the identification of fungus after periodic acid–Schiff (PAS) staining.

### *M*. *leprae* repetitive element (RLEP) PCR

All tissue sections from armadillos were investigated for the presence of *M*. *leprae* DNA by quantitative PCR (qPCR). DNeasy Blood and Tissue kit (Qiagen, Valencia, CA, USA) were used for the DNA extraction. The qPCR detection of the *M*. *leprae* repetitive element (RLEP) gene sequence used the following pair of primers (sense 5_ATTTCTGCCGCTGGTATCGGT 3_, antisense 5_TGCGCTA-GAAGGTTGCCGTAT 3_) (Thermo Fisher Scientific, Waltham, MA, USA) [17]. The primers amplify a 148-bp sequence of the RLEP element. Different amounts of purified DNA from *M*. *leprae* maintained into nude mice passages (Thai 53) were added to all negative PCR samples to verify the presence of possible inhibitory substances. A standard curve was constructed by serial dilution of purified *M*. *leprae* DNA ranging from 10fg to 1μg. Purified *M*. *leprae* DNA was also used as a positive control for the amplifications.

## Results

### Leprosy survey in rural communities

**Fig 3:**
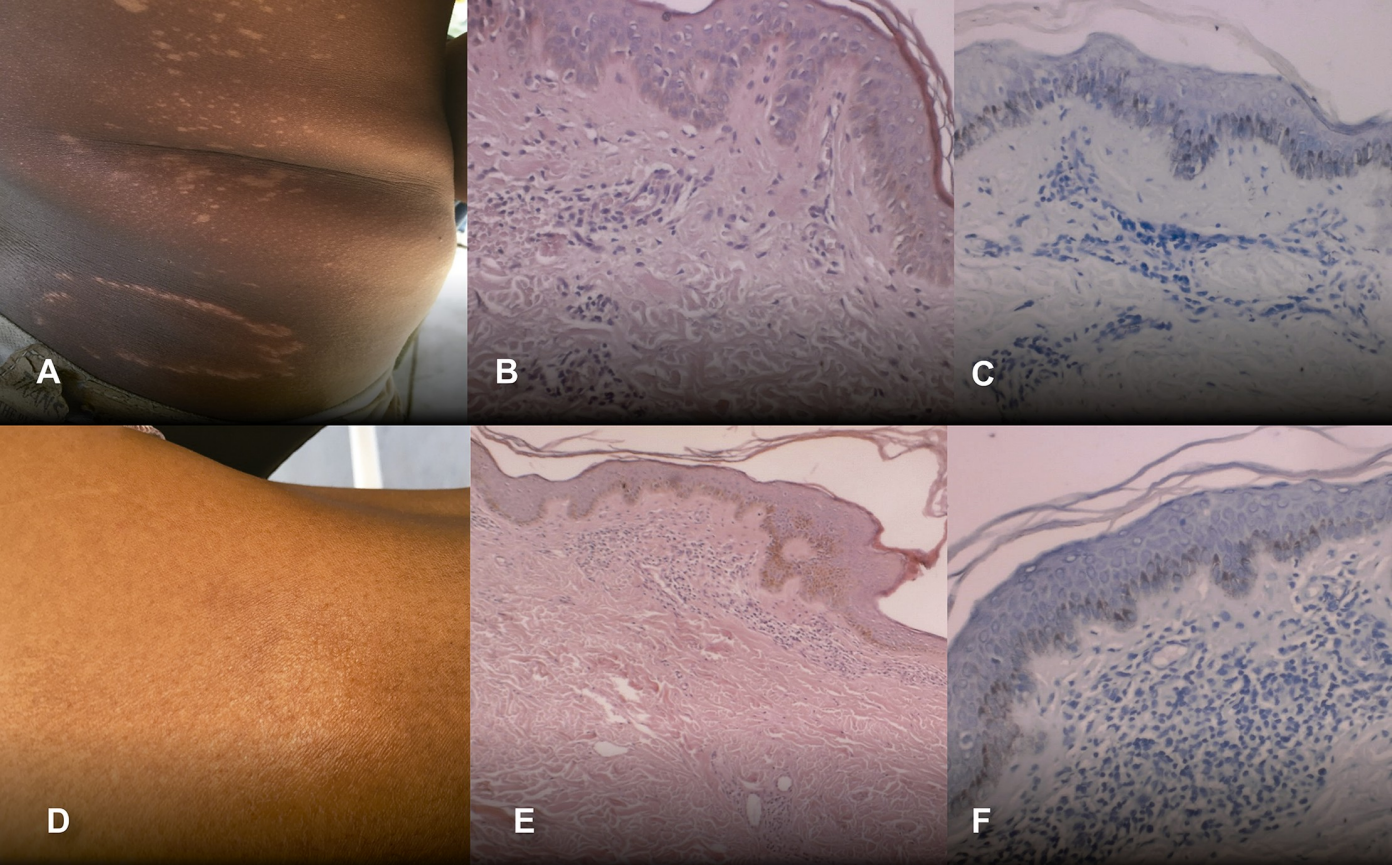
Two newly diagnosed indeterminate leprosy cases from rural communities of Coari. (A), (D): Features of skin lesions of two confirmed cases of indeterminate leprosy; (B), (E): HE staining of sections of skin biopsies of indeterminate leprosy cases showing nonspecific inflammatory infiltrates of lymphocytes and histiocytes around superficial and deep vessels and cutaneous appendages (40X); (C), (F): Negative Fite- Faraco staining for bacilli detection in sections of indeterminate leprosy skin lesions shown in (A) and (B) respectively (100x).

The screening of 176 local volunteers identified six new leprosy cases (6 out of 176; incidence = 3.4%), five were female (mean age = 27.1 years, range 18–42 years). Four of the newly diagnosed leprosy patients were from Jacaré community, one was from Xibiu and the other was from Plano de Deus community. Skin lesions of two of them are illustrated in Fig 3A and 3D. The histopathological examination of HE sections of biopsies collected from suspect skin lesions showed nonspecific inflammatory infiltrates consistent with the diagnosis of indeterminate leprosy containing lymphocytes and histiocytes around superficial and deep vessels and cutaneous appendages (Fig 3B and 3E). No bacillus was detected in these skin sections stained with Fite-Faraco (Fig 3C and 3F) or in slit skin smears (data not shown). All newly diagnosed leprosy cases received free MDT provided by the closest local public health service.

### Leprosy survey in wild armadillos

Twelve wild young adult *D*. *novemcinctus* armadillos, males and females, weighing from 4-5kg, caught in the rural area of Coari municipality were investigated for *M*. *leprae* infection.

A total of 48 biological specimens were obtained from these twelve armadillos comprising tissue fragments from skins, spleens, livers, lymph nodes, adrenal glands, ovary and fallopian tubes. From these, 96 slides were prepared for histopathology and a median number of eight slides was examined per animal (ranging from 4 to 12 slides). The detailed HE histopathology and the FF findings of each armadillo tissue examined are described in the S1 Table.

**Fig 4:**
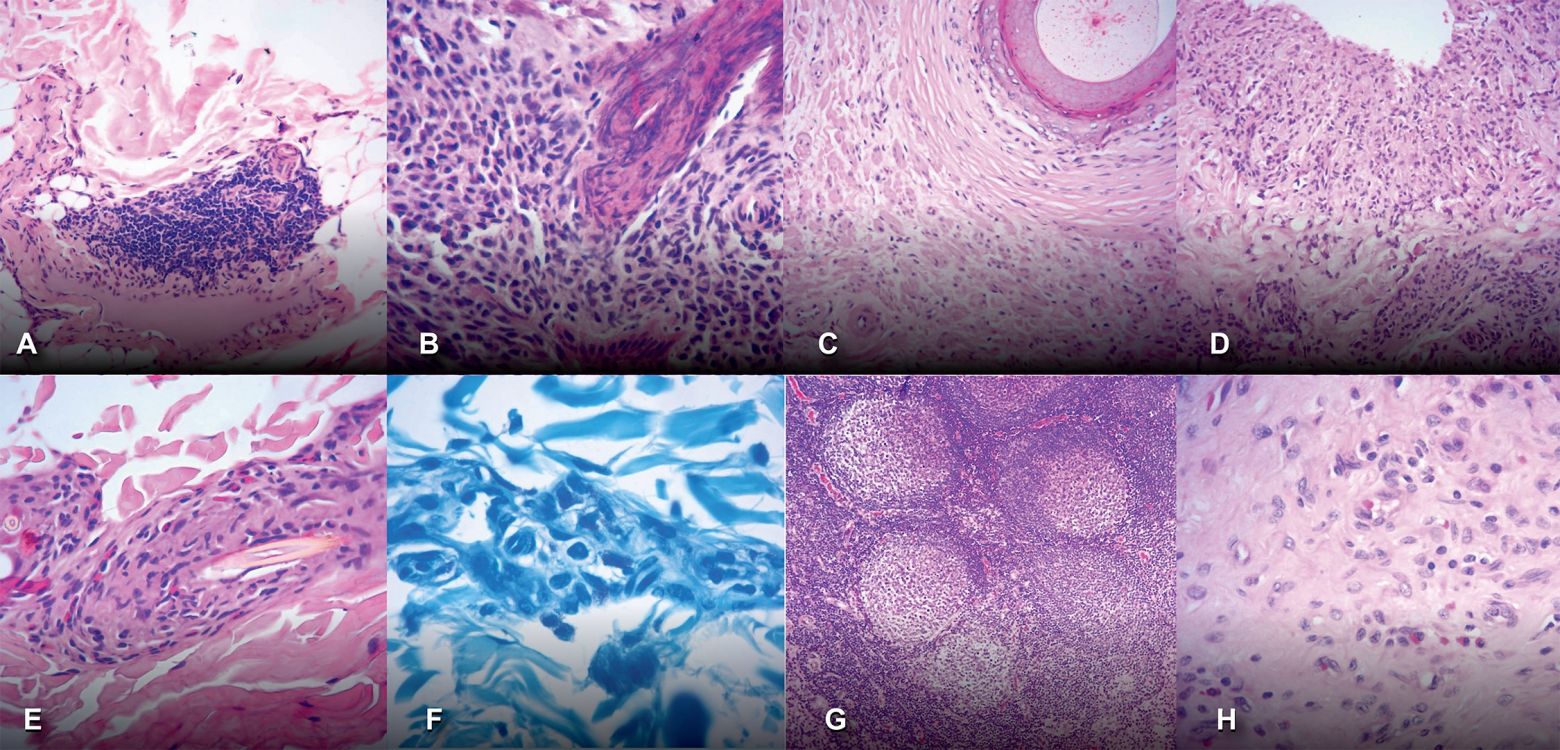
Main histopathologic findings in armadillos’ tissues. (A), (B): unspecific inflammatory infiltrate suggestive of indeterminate leprosy; (C): possible traumatic neuroma, without granuloma; (D): granuloma with epithelioid and Langhans cells; (E): foreign-body granuloma; (F): Mast cells in the skin were observed in in Fite-Faraco stained sections; (G): lymphadenitis in a lymph node section; (H) Mast cells and eosinophils infiltrating a lymph node.

Overall, the armadillo’s HE stained tissue sections examined did not show histopathological features of *M*. *leprae* infection, except for one skin fragment that presented unspecific inflammatory infiltrate suggestive of indeterminate leprosy (Fig 4A and 4B, animal #15–109). The HE histopathology showed focal chronic dermatitis extending to subcutaneous layer containing small agglomerates of perivascular mononuclear cells, preserved nerve bundles and absence of bacillus. In other armadillos, skin sections showed mild, unspecific inflammatory infiltrates. Histopathologic examinations also identified a possible traumatic neuroma, without granuloma (Fig 4C, animal #15–96), one case of granuloma with epithelioid and Langhans cells (Fig 4D, animal #15–98) and one case of foreign body granuloma, possibly secondary to ruptured hair follicle (Fig 4E, animal #15–102). Mast cells in the skin were observed in three armadillos (animals #15–103, 15–104, 15–109), (Fig 4F, animal #15–109) suggesting possible allergic reactions. Reactive lymphadenitis was seen in all lymph node sections examined (Fig 4G, animal # 15–100) and some of them contained numerous mast cells and eosinophils (Fig 4H, animal #15–96).

*M*. *leprae*- specific PCR for RLEP gene sequence was negative in all armadillo tissue samples tested. *M*. *leprae* Thai 53 DNA that was added to the negative PCR samples resulted in amplification of the RLEP amplicon in all extracts demonstrating that DNA samples from armadillos did not contain inhibitory substances. None of the armadillo’s tissue sections examined was positive for bacilli detection by Fite-Faraco. PAS staining for fungal infection was negative in skin sections presenting granulomas (animals #15–98), foreign-body granuloma (animal #15–102) and non-granulomatous chronic dermatitis (animals # 15–103, 15–104, 15–109) (data not shown).

## Discussion

In this study, we have conducted an ecological leprosy survey in a highly endemic region in the Brazilian Amazon aiming to detect new leprosy cases in rural communities as well as among wild *D*. *novemcinctus* armadillos. In sixteen rural communities surveyed, six indeterminate leprosy cases were diagnosed in volunteers and 12 wild armadillos were available for investigation. Among these armadillos, one animal showed skin histopathology compatible with indeterminate leprosy. Although another armadillo showed granulomas with epithelioid and Langhans cells, the etiology of these granulomas is only speculative. Previous studies from the Brazilian Amazon have identified armadillos infected with *Paraccocidioides brasiliensis* however in our study, none of the armadillos skin tissues with granulomatous or non-granulomatous dermatitis was positive for fungal detection [18, 19].

The possible association between the human exposure to the nine-banded armadillo and the development of leprosy has long been reported in several countries, including Brazil [20, 21]. Epidemiological studies in the US have shown contact with armadillos to be a significant risk factor for leprosy [22]. In the US autochthonous cases of leprosy in native-born citizens without any previous history of local or foreign contact with leprosy patients have been reported [23–25]. Also, in the US, there is an overlap between the areas of highest rates of autochthonous leprosy cases and of wild *D*. *novemcinctus* armadillos infected with *M*. *leprae* [5, 26]. A study in the southern US showed that the majority of US-born residents with leprosy and who referred exposure to armadillo by hunting or consuming its meat showed the same *M*. *leprae* genome sequences of the naturally infected armadillos. These strains belonged to a new *M*. *leprae* genotype (3I-2-v1), not previously reported elsewhere in the world [25]. A more recent study used *M*. *leprae* DNA PCR and specific antibodies detection in 645 armadillos from eight locations with enzootic leprosy in southeastern US [27]. *M*. *leprae* infected animals were found in each site and the *M*. *leprae* genotype 3I-2-v1 was identified in 35 armadillos. Seven other animals had a newly identified genotype (3I-2-v15). Additionally, 52 patients from the same region had 31 *M*. *leprae* types, however 42.3% of them were infected with one or two *M*. *leprae* strains associated with armadillos. This study shows that the geographic extent and the genetic diversity of zoonotic leprosy in the southeastern US is increasing.

In our study, all new leprosy cases identified in humans were considered indeterminate infections and the possible case of *M*. *leprae* infection in armadillo was also compatible with indeterminate leprosy. However, this finding was not confirmed by RLEP-PCR. Indeterminate leprosy is considered the earliest skin lesion of leprosy and is characterized by low or absent bacillary load. While the evidences gathered in this ecological study suggest that armadillos from the Brazilian Amazon could be naturally infected with *M*. *leprae*, the absence of bacilli DNA amplification, precluded the sequencing of *M*. *leprae* isolates infecting humans and armadillos, which is an essential step to establish possible links between human and armadillo disease.

Previous studies in the US have shown that 70% of the animals manifest a lepromatous-type multibacillary infection, while some animals produce paucibacillary-type infections of tuberculoid or borderline forms of the disease [28]. If armadillos present a paucibacillary leprosy infection, which could be the case of the animal identified in this study, the chances of direct microscopic identification of bacilli in tissues or by molecular approach would also be lower than in multibacillary disease. All newly diagnosed leprosy patients identified in our study area were indeterminate form, suggesting that paucibacillary disease may be frequent in this region. Therefore, in our study, the possibility of paucibacillary infection in armadillos, which could not be reliably detected by qPCR, can only be speculated.

Biomarkers of *M*. *leprae* infection in wild armadillos have been reported in Argentina, Brazil and Colombia [29, 30, 31]. In northeastern Brazil, *M*. *leprae* PCR positivity was reported in five out 15 armadillos [32] while IgM anti PGL-I ML Flow rapid test showed 11 positive armadillo serum samples out of 37 (29.7%) [30]. However, a study from southeastern and central western Brazil did not find *M*. *leprae* infection in 44 armadillos from four different species, including *D*. *novemcinctus* [33]. A recent study conducted in the Brazilian Amazon showed the presence of the *M*. *leprae*- RLEP in spleen and liver samples from 62% of the armadillos (10/16). Immunohistochemistry of spleen sections of infected animals showed in situ mycobacterial DNA, cell wall constituents and *M*. *leprae*-PGL-I antigen [16]. Although Pará and Amazonas are neighboring states in the Brazilian Amazon, no ecologic or geographical barrier that could restrict movement of armadillos between these two states exist. In a straight line, the approximate distance between the study areas (Coari and Belterra cities located in Amazonas and Para states respectively) is around 1,000 km, however there is no road between them. To reach Coari departing from Belterra (and also for the great majority of cities within Amazonas state), the only transportation route is fluvial, which is often tortuous, significantly increasing the distance between these cities. Despite these apparently conflicting findings, Brazil is a large country with significant socio demographic disparities, diverse living conditions and different leprosy prevalence and studies about the prevalence of *M*. *leprae* infection in wild armadillos are still scarce. It is possible that different endemicity levels between our study area and Belterra in Pará may account for the different findings. Also, it should be considered that, as all wild animals, armadillos are highly out-bred and may show wide differences in response to *M*. *leprae* infection [34].

Armadillo-to-armadillo transmission of *M*. *leprae* can occur via aerosol droplets through direct contact during mating or aggressive interactions, or through indirect interaction with contaminated soils during foraging [5]. It has been reported that prevalence rates in animal populations depend in the season and in the local variation in population density or population structure, both of them can affect detectability of the pathogen [35] In southern US, inter-armadillo transfer of *M*. *leprae* infection appears to be highly efficient, since the new *M*. *leprae* genotype (3I-2-v1) was found infecting armadillos across five US States [25]. A recent study in armadillos showed that the zoonotic *M*. *leprae* strain 3I does not have any growth advantage compared to the genetically distant strain SNP type-4P. The proliferation of the 4P strain was higher than the growth of the 3I strain in individually infected and in the 3I-4P co-infected armadillos suggesting pathological differences between these two strains [36]. We acknowledge that the detection of naturally infected animals in a small sample size, as our animal population investigated, would require a relatively high prevalence in order to reliably detect the infection [27]. Indeed, the environmental regulations regarding hunting protected wild animals and a concern about external visitors investigating the possible presence of an infectious organism in these animals, have probably limited the availability of a larger sample size in our study.

## Conclusions

Our survey in rural communities of Coari city, in the Brazilian Amazon confirmed high endemicity for leprosy while out of twelve armadillos just one was compatible with paucibacillary *M*. *leprae* infection. The role that armadillos may have in perpetuating leprosy in the Americas remains to be elucidated. However, in the highly endemic rural area of Coari municipality in the Amazon region, where infectious sources from untreated multibacillary leprosy are abundant, human-to-armadillo or armadillo-to-human transmission of *M*. *leprae* may not represent a major source of infection nor a significant public health concern.

## Supporting information

S1 Table Main histopathologic findings in armadillos’ tissue sections. HE: Hematoxilin-Eosin staining; AFB: acid fast bacilli detected after Fite Faraco staining; PAS: periodic acid–Schiff staining for fungus.
(DOCX)
