# Supplementary material for: Leprosy survey among rural communities and wild armadillos from Amazonas state, Northern Brazil
Source: PLoS One. 2019 Jan 10;14(1):e0209491. doi: 10.1371/journal.pone.0209491 (PMC6328080; doi:10.1371/journal.pone.0209491)
Supplement: S1 Table — HE: Hematoxilin-Eosin staining; AFB: acid fast bacilli detected after Fite Faraco staining; PAS: periodic acid–Schiff staining for fungus. (DOCX) [file pone.0209491.s001.docx]

**S1 Table: Main histopathologic findings in armadillos’ tissue sections.**

| **Animal** | **# 15-95** |
| --- | --- |
| **Organ** | **Description** |
| **Skin** | Normal skin, free neural bundles, absence of AFB. |
| **Skin** | Normal skin, preserved neural bundles, absence of AFB. |
| **Skin** | Normal skin, free neural bundles, absence of AFB. |
| **Lymph node** | Reactional lymphadenitis, hyperplasic lymphoid follicles, prominent germinative centers of different sizes. Many mast cells, eosinophils. Free neural bundles, absence of AFB. |
| **Number of slides examined**: | 08 - HE and Fite-Faraco |
| **Animal** | **# 15-96** |
| **Organ** | **Description** |
| **Skin** | Normal skin, preserved neural bundles, absence of AFB |
| **Spleen** | Reactive spleen. |
| **Liver** | Normal tissue, absence of AFB. |
| **Adrenal gland** | Normal tissue, absence of granuloma and of AFB. |
| **Skin** | Preserved neural bundles, area suggestive of traumatic neuroma, absence of granuloma and of AFB. |
| **Lymph node** | Reactional lymphadenitis, hyperplasic lymphoid follicles, exuberant germinative centers of different sizes. Many mast cells, eosinophils. Free neural bundles, absence of AFB. |
| **Number of slides examined**: | 12 - HE and Fite-Faraco |
| **Animal** | **# 15-97** |
| **Organ** | **Description** |
| **Skin** | Normal skin, preserved neural bundles, absence of AFB |
| **Lymph node** | Remarkable reactional lymphadenitis, hyperplasic lymphoid follicles with large germinative centers of different sizes. Several mast cells, eosinophils, histiocytes (epitheloid cells?). Preserved neural bundles, absence of AFB. |
| **Liver** | Normal tissue, absence of AFB. |
| **Skin** | Area with chronic inflammatory non-granulomatous process with perivascular eosinophils. Mild perineural infiltrate. Absence of AFB. PAS negative for fungus. |
| **Number of slides examined**: | 08 - HE and Fite-Faraco |
| **Animal** | **#15-98** |
| **Skin** | Chronic inflammatory granulomatous process with epithelioid and giant Langhans cells; absence of AFB |
| **Skin** | Perivascular superficial chronic dermatitis. Preserved neural bundles, absence of AFB. |
| **Lymph**  **node** | Remarkable reactional lymphadenitis, hyperplasic lymphoid follicles with large germinative centers of different sizes. Many mast cells and large histiocytes. |
| **Adrenal gland** | Normal tissue; absence of granuloma and absence of AFB. |
| **Number of slides examined**: | 08 - HE and Fite-Faraco |
| **Animal** | **#15-99** |
| **Organ** | **Description** |
| **Skin** | Normal skin, preserved neural bundles; absence of AFB. |
| **Adrenal gland** | Normal tissue, absence of granulomas and of AFB. |
| **Skin** | Perivascular superficial chronic dermatitis, preserved neural bundles, absence of AFB. |
| **Ovary and uterine tube** | Normal tissue, preserved neural bundles, absence of AFB. |
| **Number of slides examined**: | 08 - HE and Fite-Faraco |
| **Animal** | **#15-100** |
| **Organ** | **Description** |
| **Skin** | Normal skin, preserved neural bundles, absence of AFB. |
| **Lymph node** | Remarkable reactional lymphadenitis, hyperplasic lymphoid follicles with large germinative centers of different sizes. Several mast cells and large histiocytes. Preserved neural bundles, absence of AFB. |
| **Skin** | Focal peri-follicular superficial chronic dermatitis, preserved neural bundles, absence of AFB. |
| **Number of slides examined**: | 06 - HE and Fite-Faraco |
| **Animal** | **#15-101** |
| **Organ** | **Description** |
| **Skin** | Mild chronic superficial dermatitis, preserved neural bundles, absence of AFB. |
| **Liver** | Mild increase of mononuclear inflammatory infiltrate, absence of AFB. |
| **Skin** | Normal skin, preserved neural bundles, absence of AFB. |
| **Lymph node** | Reactional lymphadenitis, hyperplasic lymphoid follicles with germinative centers of different sizes. Absence of AFB. |
| **Number of slides examined**: | 08 - HE and Fite-Faraco |
| **Animal** | **#15-102** |
| **Organ** | **Description** |
| **Skin** | Foreign-body-like focal chronic granulomatous dermatitis, follicles debris, preserved neural bundles; absence of AFB. |
| **Lymph**  **node** | Reactional lymphadenitis, hyperplasic lymphoid follicles with germinative centers of different sizes. |
| **Liver** | Mild mononuclear inflammatory infiltrate in the portal space, absence of AFB. |
| **Adrenal gland** | Autolysis and vacuolar degeneration of intermediary medullar layers, absence of granulomas and of AFB. |
| **Number of slides examined**: | 08 - HE and Fite Faraco |
| **Animal** | **#15-103** |
| **Organ** | **Description** |
| **Skin** | Normal skin, preserved neural bundles, absence of AFB. |
| **Skin** | Non-granulomatous focal chronic dermatitis extending to subcutaneous tissue. Absence of AFB. Small clusters of mononuclear cells. Mast cells. |
| **Number of slides examined**: | 04 - HE and Fite-Faraco |
| **Animal** | **#15-104** |
| **Organ** | **Description** |
| **Lymph node** | Reactional lymphadenitis, hyperplasic lymphoid follicles with germinative centers of different sizes. Sinus histiocytosis. Eosinophils. |
| **Skin** | Non-granulomatous focal chronic dermatitis. Mast cells. Preserved neural bundles, absence of AFB. |
| **Number of slides examined**: | 06 - HE and Fite-Faraco |
| **Animal** | **#15-108** |
| **Organ** | **Description** |
| **Skin** | Normal skin, preserved neural bundles, absence of AFB. |
| **Skin** | Normal skin, preserved neural bundles, absence of AFB. |
| **Liver** | Normal tissue, preserved neural bundles, absence of AFB. |
| **undefined** | Tubular structure with tubules and papillae. Simple columnar epitelial lining. |
| **Adrenal gland** | Normal tissue, absence of granuloma, absence of AFB. |
| **Number of slides examined**: | 10 - HE and Fite-Faraco |
| **Animal** | **#15-109** |
| **Organ** | **Description** |
| **Skin** | Normal skin, preserved neural bundles, absence of AFB. |
| **Skin** | Non-granulomatous focal chronic dermatitis extending to subcutaneous tissue. Preserved neural bundles. Absence of AFB. Small clusters of mononuclear cells. Many mast cells. |
| **Adrenal gland** | Normal tissue. Absence of granuloma and of AFB. Preserved neural bundles. |
| **Liver** | Normal tissue. Absence of AFB. Preserved central lobular veins. |
| **Spleen** | Reactive spleen. |
| **Number of slides examined**: | 10 - HE and Fite-Faraco |
